# Invasive surgery reduces infarct size and preserves cardiac function in a porcine model of myocardial infarction

**DOI:** 10.1111/jcmm.12656

**Published:** 2015-08-18

**Authors:** Gerardus PJ van Hout, Michel PJ Teuben, Marjolein Heeres, Steven de Maat, Renate de Jong, Coen Maas, Lisanne HJA Kouwenberg, Leo Koenderman, Wouter W van Solinge, Saskia CA de Jager, Gerard Pasterkamp, Imo E Hoefer

**Affiliations:** a Experimental Cardiology Laboratory, University Medical Center UtrechtUtrecht, The Netherlands; b Department of Respiratory Medicine, University Medical Center UtrechtUtrecht, The Netherlands; c Department of Clinical Chemistry and Hematology, University Medical Center UtrechtUtrecht, The Netherlands; d Department of Anesthesiology, Erasmus Medical CenterRotterdam, The Netherlands

**Keywords:** myocardial infarction, infarct size reduction, cardioprotection, invasive surgery, large animal models

## Abstract

Reperfusion injury following myocardial infarction (MI) increases infarct size (IS) and deteriorates cardiac function. Cardioprotective strategies in large animal MI models often failed in clinical trials, suggesting translational failure. Experimentally, MI is induced artificially and the effect of the experimental procedures may influence outcome and thus clinical applicability. The aim of this study was to investigate if invasive surgery, as in the common open chest MI model affects IS and cardiac function. Twenty female landrace pigs were subjected to MI by transluminal balloon occlusion. In 10 of 20 pigs, balloon occlusion was preceded by invasive surgery (medial sternotomy). After 72 hrs, pigs were subjected to echocardiography and Evans blue/triphenyl tetrazoliumchloride double staining to determine IS and area at risk. Quantification of IS showed a significant IS reduction in the open chest group compared to the closed chest group (IS *versus* area at risk: 50.9 ± 5.4% *versus* 69.9 ± 3.4%, *P* = 0.007). End systolic LV volume and LV ejection fraction measured by echocardiography at follow-up differed significantly between both groups (51 ± 5 ml *versus* 65 ± 3 ml, *P* = 0.033; 47.5 ± 2.6% *versus* 38.8 ± 1.2%, *P* = 0.005). The inflammatory response in the damaged myocardium did not differ between groups. This study indicates that invasive surgery reduces IS and preserves cardiac function in a porcine MI model. Future studies need to elucidate the effect of infarct induction technique on the efficacy of pharmacological therapies in large animal cardioprotection studies.

## Introduction

Myocardial infarction (MI) and heart failure (HF) remain the most important cardiovascular causes of death worldwide 1,2. Because of improved medical care and revascularization therapy, survival after MI has increased considerably during the past decades 3,4. This improved survival increases the risk to develop HF as patients survive with a severely deteriorated cardiac function 1. To prevent progression into HF and preserve cardiac function post-MI, investigators aim to modulate the predominant mechanisms involved in MI and post-MI healing (reperfusion injury, adverse cardiac remodelling) 5,6.

Before clinical testing of therapeutics is considered, thorough testing in clinically relevant animal models should be performed 7. In this perspective, large animal MI models are required to confirm findings from studies performed in small animals, as the development of tissue damage post-MI differs in small animals compared to larger mammals 8,9. Additionally, large animal models show great similarity with humans regarding hemodynamics, cardiac anatomy (coronary) pharmacokinetics and –dynamics 10,11. Large animal models also allow the use of clinical treatment regimens and administration routes along with identical function-related read-outs 12,13. Despite these advantages, many compounds that were successful in large animal models eventually failed in clinical trials, suggesting optimization of experimental protocols remains necessary 14.

Numerous methods of inducing myocardial ischemia in large animals have been proposed. Despite abundant variations of ischemia induction, there are two main approaches 11. The first requires invasive surgery, including lateral or medial sternotomy, to reach the coronary arteries and induce ischemia from an external approach. The second method mimics percutaneous coronary intervention and follows a transluminal approach. The invasive method induces bone trauma and alters intra-thoracic pressure, possibly influencing cardiac physiology whereas trauma induced by the minimal-invasive method is much less extensive 11–13,15.

Previous reports from small and large animal studies indicate that abdominal skin injury prior to MI limits infarct size (IS) after cardiac ischemia 16,17. It has also been established that tissue damage, as can be observed during MI and traumatic injury, attracts circulating leucocytes to the site of injury 18–20. Furthermore, surgery can cause a decreased *ex vivo* responsiveness of leucocytes 21,22. As additional injury is known to influence myocardial IS and temporary immune suppression occurs in patients undergoing surgery, we hypothesized that medial sternotomy required for external MI induction decreases myocardial IS and preserves cardiac function post-MI. Additionally, we aimed to determine if sternotomy influences the local inflammatory response in the myocardium. This may have significant implications for the interpretation of experimental studies on therapeutic compounds tested in either model.

## Material and methods

All animal experiments were approved by the institutional animal welfare committee of the University Medical Center Utrecht and were executed conforming to the ‘Guide for the Care and Use of Laboratory Animals’. Twenty female landrace pigs were evaluated in this study (Van Beek, Lelystad, The Netherlands). Pigs (69.8 ± 1.0 kg) were subjected to MI by transluminal balloon occlusion of the left anterior descending artery (LAD) followed by invasive pressure-volume measurements; 3D-Echocardiography and Evans blue/triphenyl tetrazoliumchloride (TTC) double staining to determine IS and area at risk (AAR) at 72 hrs follow-up. In 10 of 20 pigs, balloon-occlusion was preceded by medial sternotomy.

### Surgical procedure

Pre-treatment and anaesthesia protocols have been described elsewhere 12,23. In short, all animals were pre-treated with amiodaron for 10 days (1200 mg loading dose, 800 mg/day maintenance), clopidogrel for 3 days (75 mg/day) and acetylsalicylic acid for 1 day (320 mg loading dose, 80 mg/day maintenance). All medication was continued until the end of the study. To prevent unnecessary stress and discomfort, animals were anaesthetized with an intramuscular injection of 10 mg/kg ketamine, 0.4 mg/kg midazolam and 0.014 mg/kg atropine. Venous access was obtained by insertion of an 18G cannula in the ear vein for intravenous administration of 5 mg/kg sodiumthiopental. Depth of anaesthesia was then determined by checking eyelid reflex, response to skin stimulus and laryngeal reflex followed by intubation with an endotracheal tube and balloon-ventilation. Pigs were transported to the operating room where anaesthesia was maintained by intravenous infusion of 0.5 mg/kg/hr midazolam, 2.5 μg/kg/hr sufentanyl and 0.1 mg/kg/hr pancuronium. Pigs were mechanically ventilated and arterial blood pressure and heart rate were checked when performing surgical actions to determine the depth of anaesthesia. Pre-operatively, animals received a fentanyl patch (25 μg/hr). Arterial access was obtained by introduction of an 8F sheath into the carotid artery after surgical exposure. Pigs were then randomly allocated to either an open or closed chest procedure.

### Closed chest group

After arterial and venous access were obtained, the closed chest animals were left untouched for 20 min. This corresponds to the time required to perform the sternotomy in the open chest group. A coronary angiogram of the left coronary tree was acquired using an 8F JL4 guiding catheter (Boston Scientific, Natick, MA, USA). An adequately sized balloon was placed distal to the second diagonal branch and inflated for 75 min. After reperfusion, a catheter was temporarily placed in the coronary sinus *via* an introducer sheath in the jugular vein to draw blood 60 min. after reperfusion. Animals were observed for 3 hrs post-reperfusion and blood samples were drawn at various time-points. A dwelling catheter was placed in the jugular vein in 3 pigs of each experimental group to allow additional venous blood sampling at 4 and 8 hrs reperfusion. The surgical wound was closed and animals were weaned from anaesthesia. Animals were defibrillated in case of ventricular fibrillation (VF). Body temperature was kept constant between 37.0 and 38.5°C throughout the experiment. Heart rate and arterial blood pressure were measured continuously and documented every 30 min.

### Open chest group

The open chest group was treated identical to the closed chest group with the exception that in the 20 min. period after the insertion of the arterial and venous sheaths, a medial sternotomy was performed. After performing the myocardial ischemia/reperfusion protocol as described above, the sternum and the surgical wound were closed and animals were weaned from anaesthesia.

### Pressure–volume measurements

Pressure–volume measurements were performed as described recently 23,24. In short, animals were again anaesthetized 72 hrs after infarct induction. Arterial access was obtained by introducing an 8F sheath into the carotid artery. A 7F tetra-polar admittance catheter (7.0 VSL Pigtail/no lumen; Transonic Scisense, London, ON, Canada) was inserted into the left ventricle (LV) through the sheath in the carotid artery under fluoroscopic guidance.

### Echocardiography

Three-dimensional echocardiography was performed as described before 12,24,25. In short, an X3-1 transducer on an iE33 ultrasound device (Philips, Eindhoven, The Netherlands) was used 72 hrs after reperfusion. Immediately after PV measurements, a medial (re)sternotomy was performed and a gel-filled flexible sleeve was placed directly on the apex of the heart. The depth and sector size were adjusted to fit the complete ventricle. The images were analysed offline using QLab 10.1 (Philips, Eindhoven, The Netherlands) (3DQ advanced) analysis software.

### Area at risk and infarct size

Before exsanguination, the leukocyte adhesion deficiency (LAD) was externally ligated at the exact site of balloon occlusion. The LV was punctured with a sterile needle attached to a 50 ml syringe filled with 2% Evans blue dissolved in 50 ml 0.9% NaCl. The aortic root was clamped distal to the origin of the coronary arteries. Evans blue was infused at a rate of 10 ml/sec. Animals were then killed by exsanguination under anaesthesia. The heart was excised and the LV was cut into 5 equal slices from apex to base. Slices were incubated in 1% TTC (Sigma-Aldrich Chemicals, Zwijndrecht, The Netherlands) in 37°C 0.9% NaCl for 10 min. to discriminate between infarct tissue and viable myocardium. After incubation, photographs of the slices were taken at the remote area, AAR and infarcted tissue were quantified using ImageJ software (NIH, Bethesda, MD, USA). Following quantification, infarcted tissue, tissue from the border zone and tissue from the remote area were collected and either conserved in 4% formaldehyde for histological quantification of neutrophils, or snap frozen in liquid nitrogen for cytokine measurements.

### Neutrophil numbers, macrophage numbers and inflammatory parameters

Circulating leucocyte numbers from blood drawn at multiple time-points after reperfusion were measured by whole-blood analysis using an automated haematological cell-counter (Cell-Dyn Sapphire; Abbott, Santa Clara, CA, USA). The Cell-Dyn Sapphire is a routine haematology analyzer, which uses spectophotometry, electrical impedance and laser light scattering to classify blood cells (platelets, erythrocytes and leukocytes).

Neutrophil numbers, macrophage numbers and active caspase-3 positive cells in myocardial tissue were measured in paraffin-embedded histological biopsies that were conserved in 4% formaldehyde for at least 7 days. Histological samples were cut into 5 μm sections using a microtome and sections were deparaffinized. To assess neutrophil numbers, sections were incubated with a porcine-specific monoclonal mouse anti-pig antibody against porcine neutrophils (Clone PM1; BMA Biomedicals, Augst, Switzerland) for 60 min. followed by incubation with Brightvision Poly-AP-antimouse (ImmunoLogic, Duiven, The Netherlands) for 30 min. To determine macrophage numbers, sections were incubated with a monoclonal mouse anti-pig CD107a antibody (Serotec, Raleigh, NC, USA) followed by incubation with the same secondary antibody. Caspase-3 positive cells were assessed using a purified rabbit anti-active caspase-3 antibody (BD Pharmigen, San Diego, CA, USA) followed by incubation with Brightvision Poly-AP-anti-rabbit (Immunologic) for 30 min. For microscopic visualization, incubation with liquid permanent red (DAKO, Heverlee, Belgium) for 10 min. was performed. For every animal, 10 random pictures were made at 200× magnification and quantified using CellSens software (Center Valley, PA, USA).

Malondialdehyde (MDA) is a marker of lipid peroxidation and oxidative stress and was measured to determine if sternotomy induced less oxidative stress in the myocardium during reperfusion. Malondialdehyde was measured from plasma obtained by centrifugation at 1850 × g for 10 min. at 4°C. To quantify MDA, we used a lipid peroxidation – MDA – kit (Abcam, Cambridge, MA, USA) according to the manufacturer's instructions.

To assess the inflammatory response in the myocardium, the expression of 9 different cytokines [interleukin (IL)-1β, IL-4, IL-6, IL-8, IL-10, IL-12p40, tumour necrosis factor (TNF)-α, interferon (IFN)-α and IFN-γ] was measured using a luminex immunoassay (Procarta^™^ Multiplex; eBioscience, San Diego, CA, USA) according to the manufacturer's instructions.

### Western blotting

High molecular weight kininogen (HMWK) was measured as a proxy of bradykinin release. Plasma samples drawn at baseline and 15 min. reperfusion were diluted 20 times in reducing sample buffer (15.5% Glycerol, 96.8 mM Tris-HCL, 3.1% SDS, 0.003% bromophenol blue, 25 mM Dithiothreitol) and incubated for 10 min. at 95°C. Samples were separated on a 4–12% Bis-Tris gel at 165 V for 65 min. in 3-(*N*-morpholino)propanesulfonic acid buffer. Proteins were transferred onto Immobilon-FL membranes at 125 V for 60 min. in blotting buffer (14.4 g/l glycine, 3.03 g/l Tris-HCL, 20% ethanol) and blocked with blocking buffer (0.5× Odyssey blocking reagent in Tris-buffered saline) for 1 hr at RT. High molecular weight kininogen was detected by overnight incubation at 4°C with polyclonal affinity purified sheep anti-human HMWK antibody (Affinity Biologicals Inc., Ancaster, ON, Canada). Blots were washed with 0.005% TBS-Tween (TBST), and primary antibodies were detected with Alexa Fluor^®^ 680 Donkey Anti-Sheep IgG (1:7500 in blocking buffer; Life Technologies, Carlsbad, CA, USA). Blots were extensively washed with TBST, followed by washing with water and analysed on a near infra-red Odyssey scanner (LI-COR Biotechnology, Lincoln, NE, USA).

### Statistical analysis

All data are expressed as mean ± SE unless stated otherwise. Differences in mortality were tested using a Fisher's exact test. Myocardial IS, LV ejection fraction (LVEF) and all other outcome were compared using a Student's *t*-test for unrelated measurements. All statistical analyses were performed in SPSS statistics version 20.0 (IBM software, Armonk, NY, USA). A two-sided *P*-value of <0.05 was regarded statistically significant in all analyses.

## Results

### Mortality and hemodynamics

Twenty pigs were subjected to 75 min. myocardial ischemia-reperfusion injury. Four pigs died before the follow-up period (1 in the closed chest and 3 in the open chest group, all because of the persistent VF during cardiac ischemia). Fisher's exact testing showed that this difference was statistically not significant (*P* = 0.292). This allowed a comparison of nine animals in the closed chest and seven animals in the open chest group.

**Figure 1:**
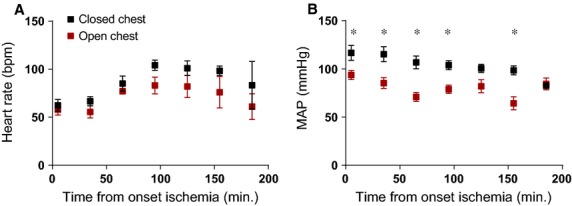
Hemodynamic parameters were recorded every 30 min. during the experiments. (**A**) Heart rate did not differ significantly from the onset of ischemia to 180 min. post-reperfusion. (**B**) A significant decrease in MAP was observed at multiple time-points during ischemia and reperfusion, peaking at 60 min. of ischemia. Bpm: beats per minute; MAP: mean arterial pressure, *P < 0.05.

During the 3-hr observation period post-MI, heart rate did not significantly differ between both groups (Fig.1A). However, from the onset of ischemia onwards, mean arterial pressure (MAP) in the open chest group was significantly lower than in the closed chest group (Fig.1B). This difference in MAP reached its peak at 60 min. post-occlusion (71 mmHg *versus* 107 mmHg, *P* = 0.006) and gradually returned to equal levels by the end of the observational period of 180 min. (85 mmHg *versus* 84 mmHg, *P* = ns).

### Myocardial infarct size and cardiac function

**Figure 2:**
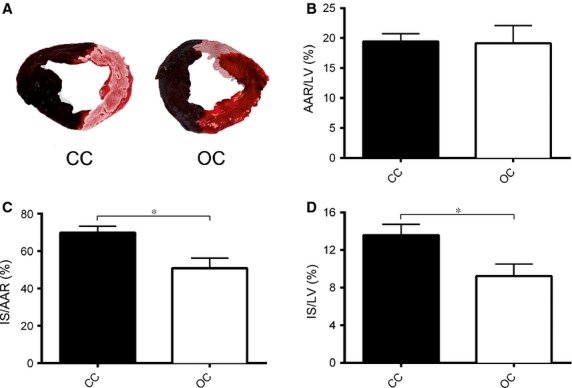
After 72 hrs of reperfusion, the AAR and IS were determined. (**A**) Representative pictures of an apical slice of a pig that was subjected to MI without (CC) and with sternotomy (OC). (**B**) The AAR as a percentage of the total LV was equal between the closed chest and open chest group. (**C**) The IS as a percentage of the AAR differed significantly between the closed chest and open chest group. (**D**) The IS as a percentage of the total LV differed significantly between the closed chest and open chest group. CC: closed chest; OC: open chest; AAR: area at risk; IS: infarct size; LV: left ventricle, *P < 0.05.

Cardiac function and myocardial IS were determined after a follow-up period of 72 hrs. Figure2A shows representative pictures of the remote area (blue), AAR (red) and infarcted myocardium (white). Quantification of the AAR showed a similar AAR as a percentage of the LV in the open chest and closed chest group (19.1 ± 3.0% *versus* 19.4 ± 1.3%, *P* = ns; Fig.2B). Quantification of IS showed a significant reduction in the open chest group compared to the closed chest group when measured as percentage of the AAR (50.9 ± 5.4% *versus* 69.9 ± 3.4%, *P* = 0.007; Fig.2C) and using the total LV as reference (9.2 ± 1.3% *versus* 13.6 ± 1.2%, *P* = 0.024; Fig.2D).

**Figure 3:**
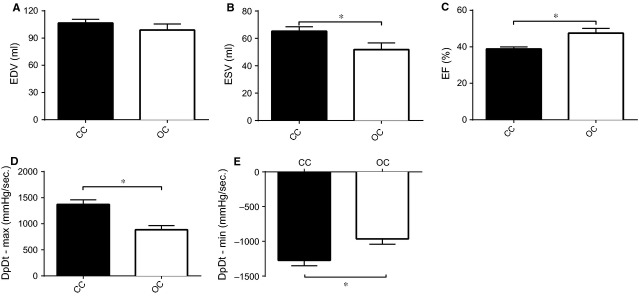
LV function was measured 72 hrs after reperfusion. (**A**) End diastolic volume was not significantly lower in pigs subjected to medial sternotomy. (**B**) End systolic volume was significantly lower in pigs subjected to medial sternotomy. (**C**) Ejection fraction was significantly higher in the open chest compared to the closed chest group. (**D**) dPdt-max was lower in de open chest group. (**E**) dPdt-min was less negative in the open chest compared to the closed chest group. EDV: end diastolic volume; ESV: end systolic volume; EF: LV ejection fraction; ml: millilitre; dPdt-max: rate in pressure change over time during systole; dPdt-min: rate in pressure change over time during diastole; CC: closed chest; OC: open chest, *P < 0.05.

**Figure 4:**
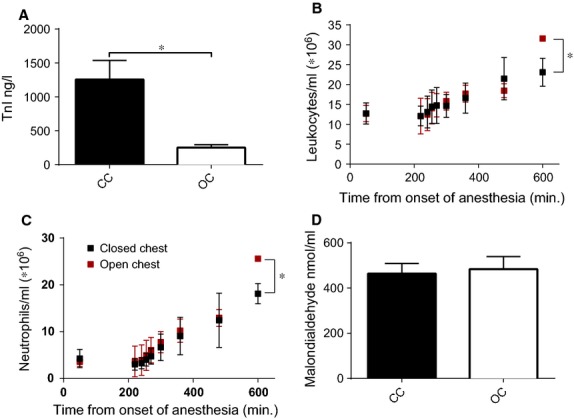
Different measurements were performed in the systemic circulation. (**A**) Troponin was significantly higher in the closed chest group compared to open chest group. (**B**) Systemic leucocyte numbers were measured at baseline and different time-points during ischemia and reperfusion. (**C**) Systemic neutrophil numbers were measured at baseline and different time-points during ischemia and reperfusion. (**D**) Lipid peroxidation measured by malondialdehyde concentration in coronary sinus blood was not different between the open chest and closed chest group. TnI: troponin I; ng: nanogram; l: litre; ml: millilitre; nmol: nanomol; CC: closed chest; OC: open chest, *P < 0.05.

Cardiac function also differed between the two experimental groups. While end diastolic volume did not differ between the open chest and the closed chest group (99 ± 7 ml *versus* 106 ± 4 ml, *P* = ns), end systolic volume (51 ± 5 ml *versus* 65 ± 3 ml, *P* = 0.033) and LVEF (47.5 ± 2.6% *versus* 38.8 ± 1.2%, *P* = 0.005) differed significantly between both groups (Fig.3A–C). Interestingly, the load-dependent rate of pressure increase (dPdt-Max) during systole was lower in the open chest group than in the closed chest group despite a smaller IS and better global cardiac function (886 ± 80 mmHg/sec. *versus* 1373 ± 87 mmHg/sec., *P* = 0.002). The load-dependent rate of pressure decrease (dPdt-Min) during diastole was less negative in the open chest group compared to the closed chest group (−964 ± 78 mmHg/sec. *versus* −1273 ± 77 mmHg/sec., *P* = 0.018; Fig.3D and E). Troponin was measured 2 hrs after reperfusion and was significantly lower in the open chest compared to the closed chest group (251 ± 44 ng/ml *versus* 1256 ± 281 ng/ml, *P* = 0.008; Fig.4A).

### Inflammatory markers, oxidative stress and bradykinin release

To determine if the two models differed in the systemic inflammatory response post-MI, we measured systemic leucocyte numbers up to 2 hrs reperfusion. As no differences in leucocyte numbers were detected during ischemia and the first 2 hrs of reperfusion (Fig.4B and C), we also measured systemic leucocyte numbers 4 and 8 hrs post-reperfusion in a subset of pigs (*n* = 3 in each group). In the open chest group, circulating leucocyte numbers were significantly higher at 8 hrs reperfusion in the open chest compared to the closed chest group (31.6 ± 0.2 × 10^6^ *versus* 23.1 ± 2.0 × 10^6^ leucocytes/ml, *P* = 0.014; Fig.4B). Neutrophil numbers were also significantly increased in this group (25.6 ± 0.1 × 10^6^ neutrophils/ml *versus* 18.1 ± 1.2 × 10^6^ neutrophils/ml, *P* = 0.040; Fig.4C) 8 hrs post-reperfusion. Other leucocyte subtypes did not differ significantly between both groups at any time-point (data not shown). As inflammatory cells are an important source of free radical scavengers at the site of injury, MDA, a marker of oxidative stress, was measured in blood directly drawn from the coronary sinus 1 hr after reperfusion in both groups. However, there was no significant difference in MDA levels between both groups (MDA concentration: 483 ± 55 nmol/ml *versus* 465 ± 45 nmol/ml, *P* = ns; Fig.4D).

**Figure 5:**
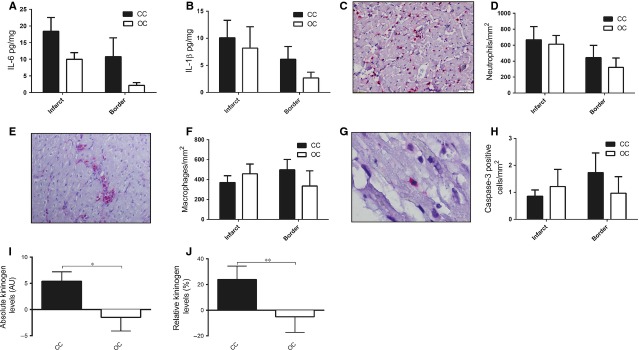
Local markers of the inflammatory response were measured in the myocardium. (**A**) Myocardial IL-6 content corrected for protein concentration in both the border and infarct zone differed non-significantly between the open chest and closed chest group. (**B**) Myocardial IL-1β content corrected for protein concentration in both the border and infarct zone differed non-significantly between the open chest and closed chest group. (**C**) Representative picture of histological section of infarcted myocardial tissue containing neutrophils (red cells) 72 hrs post-reperfusion. (**D**) Neutrophil numbers in both the border and infarcted zone of the myocardium are similar between the open chest and closed chest group. (**E**) Representative picture of macrophages (red) resided in the infarcted myocardium. (**F**) Macrophage numbers in both the border and infarcted zone of the myocardium are similar between the open chest and closed group. (**G**) Representative picture of an active caspase-3 positive cell (red). (**H**) Apoptosis of myocardial cells was similar between open chest and closed chest pigs. (**I** and **J**) Absolute and relative kininogen levels are higher in the closed chest compared to the open chest group. pg: picogram; mg: milligram; AU: arbitrary units; CC: closed chest; OC: open chest, *P < 0.05, **0.05 < *P* < 0.1.

To investigate potential differences in the severity of the local immune response, we measured a variety of cytokines known to play a role in the inflammatory phase post-MI. We did not observe any significant differences in any of the measured cytokines in the myocardium. As examples, IL-6 (infarct area, *P* = 0.15; border area *P* = 0.40) and IL-1β (infarct area, *P* = 0.9; border area *P* = 0.25) concentrations are shown in Figure5A and B. Similarly, we did not observe any significant difference between open and closed chest procedure animals regarding neutrophil and macrophage numbers in the myocardium 72 hrs after reperfusion (Fig.5C–F). Finally, we determined cardiomyocyte apoptosis 72 hrs after reperfusion. There were no significant differences between the open chest and closed chest group in caspase-3 positive cells (Fig.5G and H).

As bradykinin has previously been reported to mediate the cardioprotective effects of abdominal skin incision on reperfusion injury, we measured the bradykinin precursor HMWK. High molecular weight kininogen fragmentation serves as a proxy for bradykinin, with low HMWK levels reflecting fragmentation and high bradykinin release. Compared to baseline, absolute HMWK concentrations were higher in the closed chest compared to the open chest group (*P* = 0.043) and a trend was observed for relative expression levels between groups (*P* = 0.095), indicating that bradykinin release was higher in the open chest group (Fig.5I and J).

## Discussion

For the development and validation of novel cardioprotective strategies aiming at reduced ischemia reperfusion injury, new compounds need to be tested in models that resemble the clinical situation as closely as possible 7,26. This study indicates that medial sternotomy reduces IS and preserves cardiac function in a porcine MI model, possibly confounding the effect of pharmacological therapies in this model.

Our observation may partly be explained by the effect of open chest surgery on cardiac pressure, as MAP decreased during the first 3 hrs after cardiac ischemia. Cardiac pressure analysis over time revealed a significant decrease in load dependent dPdt-max and dPdt-min 72 hrs after surgery despite smaller IS and better cardiac function. This indicates that sternotomy unloads the LV and this effect was still measurable after 3 days. However, a reduction in myocardial workload is unlikely to be the only responsible mechanism for the observed IS reduction as previous research reported similar effects of additional trauma on IS, which persisted after blood pressure normalization by saline infusion 27.

Various small and large animal studies have shown that abdominal skin injury preceding experimental MI results in reduced IS 27,28. This observed reduction seems to be mediated through nociceptive bradykinin-mediated pathways and can be abolished by pharmacologically inhibiting these pathways 16,17,29,30. Because of its very short half-life, we were unable to detect circulating bradykinin levels in this study. However, levels of the bradykinin precursor HMWK were significantly lower in the open chest group compared to the closed chest group, possibly reflecting a higher bradykinin release. This suggests that medial sternotomy exerts its cardioprotective effects through increased bradykinin release.

A third, potentially relevant, mechanism is the inflammatory response after MI. Traumatic injury triggers a very complex immune reaction 31,32 and could therefore alter the inflammatory response that is involved in myocardial reperfusion injury and post-MI repair. However, the number of circulating leucocytes during reperfusion was not decreased in the open chest group, but actually higher at 8 hrs reperfusion. Moreover, after 72 hrs, we neither observed any significant difference in neutrophil and macrophage accumulation in the myocardium nor myocardial cytokine content, two major determinants of post-MI infarct expansion 33–35. Furthermore, no differences in cardiomyocyte apoptosis were found between the groups.

The free radical burst that occurs within the first hours after reperfusion depends on the activation of inflammatory cells during reperfusion in the damaged myocardium 20. Malondialdehyde levels in blood drawn from the coronary sinus 1 hr after reperfusion did not differ between the open chest and closed chest group. Although this suggests sternotomy does not significantly influence the inflammatory response post-MI, decreased *ex vivo* cellular responsiveness that has been observed in patients after different surgical procedures could also play a role 21,22,36.

Regardless of the mechanisms at hand, possible confounders that may obscure the effect of cardioprotective agents in studies of experimental MI should be avoided as much as possible. Better clinical resemblance of animal models will increase translational value and improve reproducibility and translatability towards clinical application.

Mortality between the 2 groups was not significantly different. However, this lack of statistical significance might be attributable to insufficient power. Mortality is an essential parameter in translational research and survival bias could be a confounder in cardioprotection studies that have high mortality rates. Unfortunately, the cause of the malignant arrhythmias in the non-surviving animals remains unknown, but something that would be worth investigating. However, in this study we were unable to clarify this issue, as we did not store any plasma or tissue of the animals that died before the follow-up time was completed.

Despite our best efforts, we could not evade introducing limiting factors ourselves. First of all, experiments were performed under general anaesthesia, which cannot be avoided in animal models. The anaesthetics used may have a protective effect on the myocardium, although the IS compared to the AAR in the minimally invasive group was reasonably high (approximately 70%). Theoretically, differences in blood loss because of the type of intervention may affect blood pressure and thus IS. However, blood loss in our experiments was minimal (<200 ml) for animals with a circulating blood volume of approximately 5 l. Furthermore, we did not find any evidence for a modulation of myocardial inflammation by medial sternotomy, indicating that the reduction in IS cannot be ascribed to this mechanism. However, to fully conclude this, follow-up studies need to be performed that specifically focus on the inflammatory response (*e.g*. other leucocyte subsets, cellular responsiveness). Finally, we did not administer any pharmacological agent to block bradykinin-mediated pathways or pro-inflammatory pathways to conclusively determine whether any of the two mechanisms could (partly) be held responsible for the IS reduction after medial sternotomy. Determining whether these or other key mechanisms play a role in the reduction in IS after medial sternotomy will allow for specifically targeting these pathways. Pharmacological inhibitors could be developed to inhibit these newly discovered cellular signals and determine whether the phenomenon that we have observed in this study, can directly be translated into clinical applications to salvage myocardium in post-MI patients. As this was not the primary purpose of this study, and therefore remains to be elucidated in future studies.

In conclusion, this study shows that medial sternotomy preceding MI in a porcine model reduces IS and preserves cardiac function. As the mechanisms responsible for this observation are not fully elucidated, it remains unclear how our findings affect the outcome of cardioprotection studies with open chest surgery. The ever-evolving techniques of percutaneous coronary interventions enable animal models to increasingly resemble the clinical situation and in combination with our results call for minimally invasive cardiac ischemia models. This may reduce the possibility of false-positive and –negative studies and improve translational value of preclinical research.
